# Bi-Monthly Abstracts

**Published:** 1858-07

**Authors:** 


BI-MONTHLY ABSTRACTS. — Under this title we propose hereafter regularly to furnish the readers of the Review with an account of the more interesting and important novelties that may reach us, from time to time, in our foreign Continental Exchanges. The department will, for the present, be under the direction of Dr. Emil Fischer, of this city, whose scholarship and judgment eminently qualify him for the position.

In consequence of the introduction of this new feature into the journal, and the large amount of original matter, our regular Periscopic selections have been excluded. They will be resumed in the September number.

